# Descriptions of Two New Species of *Encarsia* (Hymenoptera: Aphelinidae) and Mitochondrial Genome Analysis of Three Species of the Genus †

**DOI:** 10.3390/insects17030282

**Published:** 2026-03-05

**Authors:** Ye Luo, Zhigang Dong, Xiaolong Ma, Junqing Ge, Serguei V. Triapitsyn, Jian Huang, Zhuhong Wang

**Affiliations:** 1 State Key Laboratory of Agricultural and Forestry Biosecurity, College of Plant Protection, Fujian Agriculture and Forestry University, Fuzhou 350002, China; 19957242392@163.com (Y.L.); d18235546505@163.com (Z.D.); mxl19993449499@163.com (X.M.); jhuang1234@126.com (J.H.); 2 Institute of Biotechnology, Fujian Academy of Agricultural Sciences, Fuzhou 350003, China; jqge@163.com; 3 Entomology Research Museum, Department of Entomology, University of California, Riverside, CA 92521, USA; serguei@ucr.edu

**Keywords:** Aphelinidae, *Encarsia*, whitefly and scale insect, biocontrol, mitochondrial genome

## Abstract

Two new species of the genus *Encarsia* Förster (Hymenoptera: Aphelinidae), *E. cinnamomi* Wang & Huang, **sp.n.** and *E. ophiopogonis* Wang & Huang, **sp.n.,** are described and illustrated from Fujian, China. The mitochondrial genomes of these two new species and *E. diaspidicola* are sequenced (14,049 bp, 14,746 bp and 14,849 bp, respectively), both showing a strong A + T bias (84.1%, 84.7% and 84.8% respectively). We infer the phylogenies of several *Encarsia* species of Aphelinidae and other family of Chalcidoidea based on PCG123 datasets using Bayesian inference (BI) and maximum likelihood (ML) methods. ML and BI analysis both support Aphelinidae formed a sister group to Torymidae.

## Simple Summary

Species of *Encarsia* (Hymenoptera: Aphelinidae) are primary parasitoids of whiteflies and armored scale insects (Hemiptera: Aleyrodidae, Diaspididae, respectively). Several species of *Encarsia* have been successfully employed in biological control programs against agricultural pests. As the largest genus within the family Aphelinidae, *Encarsia* currently comprises 473 described species worldwide, including more than 100 species recorded in China. To date, only three mitochondrial genomes have been reported within Aphelinidae, of *Encarsia formosa*, *E. obtusiclava* and *E. agona*. Here we describe two new *Encarsia* species from Fujian, China, document their host associations, and present the mitochondrial genomes of these two new species as well as *E. diaspidicola*.

## 1. Introduction

The genus *Encarsia*, established by Förster in 1878, is a species-rich genus of the family Aphelinidae (Hymenoptera: Chalcidoidea) comprising 473 described species worldwide, including over 100 recorded species in China [1,2,3,4]. Most of *Encarsia* species are important natural enemies of armored scale insects (Hemiptera: Diaspididae) and whiteflies (Hemiptera: Aleyrodidae) that infest agricultural crops and ornamental and forestry plants, playing a crucial role in the biological control of these pests [3]. Successful examples include the introduction of *E. berlesei* (Howard) into Italy to effectively suppress the white peach scale, *Pseudaulacaspis pentagona* (Targioni-Tozzetti) [5], and the application of *E. formosa* to control whiteflies worldwide. *Encarsia formosa* has been successfully used in more than 20 countries such as the United Kingdom, the United States, the Netherlands and India, and its large-scale commercial production has been realized [6,7,8,9,10]. Also, in 1978, *E. formosa* was introduced from the United Kingdom to China to control whiteflies, and has since become an effective parasitoid against the greenhouse whitefly, *Trialeurodes vaporariorum* (Westwood), and the sweet potato whitefly, *Bemisia tabaci* (Gennadius) [9,11,12,13].

With the advancement of molecular biology research, an increasing number of molecular markers have been employed in the systematic classification of *Encarsia*. Early studies primarily relied on single-gene analyses, such as the mitochondrial genes cytochrome c oxidase subunit I (*cox1)* and cytochrome c oxidase subunit II (*cox2)* [14,15,16,17], as well as the nuclear genes 18S ribosomal DNA (18S rDNA) and 28S ribosomal DNA (28S rDNA) [17,18,19]. In recent years, the advent of high-throughput sequencing has facilitated the growing use of mitochondrial genomes in exploring phylogenetic relationships among species.

The insect mitochondrial genome is a closed circular double-stranded DNA molecule, typically ranging from 14 to 20 kb in length. It contains 37 genes: 13 protein-coding genes (PCGs), two rRNA genes (rRNAs), and 22 tRNA genes (tRNAs), along with an A + T-rich region also known as the control region or D-loop region [20,21,22]. The two strands are designated as the J-strand (majority strand), which encodes most genes, and the N-strand (minority strand), which encodes fewer genes [23]. In insects, all 13 PCGs use ATN (ATA, ATT, ATC, ATG) as the start codon and TAA or TAG as the stop codon, although a small number of genes may also use incomplete stop codons such as TA or T [24].

Due to its maternal inheritance, moderate length, and relatively stable structure, the mitochondrial genome has been widely used in species classification and phylogenetic studies [25,26]. Nevertheless, within the family Aphelinidae, mitochondrial genomic data remain scarce. To date, only two species of *Encarsia*, *E. obtusiclava* and *E. formosa*, have had their complete mitochondrial genomes reported, both of which were first described by Zhu et al. [27]. The evolutionary rate of the mitochondrial genome is commonly assessed by the ratio of non-synonymous substitution rate (Ka) to the synonymous substitution rate (Ks). A Ka/Ks value greater than 1 indicates positive selection acting on the protein-coding genes; a value equal to 1 suggests neutral evolution; and a value less than 1 reflects purifying selection [28,29].

In this study, we describe and illustrate two new species, *Encarsia cinnamomi* Wang & Huang, **sp.n.** and *E. ophiopogonis* Wang & Huang, **sp.n.**, collected from Fujian, China, and sequence and assemble the mitochondrial genomes. In addition, we sequence and assemble the mitochondrial genome of *E. diaspidicola*. Based on PCG123 (the 1st, 2nd, and 3rd codon positions of protein-coding genes) datasets using Bayesian inference (BI) and maximum likelihood (ML) methods, we infer the phylogenetic relationships of several *Encarsia* species within Aphelinidae and other families of Chalcidoidea. This work adds new mitochondrial genome data for *Encarsia*, thereby expanding the database and providing a foundation for future species identification. It also contributes to understanding the evolutionary relationships within Aphelinidae and other families of Chalcidoidea.

## 2. Materials and Methods

### 2.1. Collection of Parasitoids

Specimens of the cinnamon black fly, *Aleurocanthus cinnamomi* Takahashi (Aleyrodidae) on camphor tree, *Cinnamomum camphora* Nees ex Wall and armored scales, *Pinnaspis aspidistrae* (Signoret) (Diaspididae) on dwarf lilyturf, *Ophiopogon japonicus* (Linn.f.) Ker-Gawl were collected in Fujian Province, China. The collected specimens were placed in rearing boxes in the laboratory and kept in an artificial climate chamber set at a temperature of 26 ± 1 °C, a humidity of 65 ± 5%, and a photoperiod of 14L:10D, until the parasitic wasps emerged. *Encarsia* parasitoids reared from these hosts were preserved in 100% ethanol after emergence.

### 2.2. Photographs, Slides of Parasitoids and Morphological Identification

Prior to slide-mounting, the body color of each specimen was described and photographed. The slide-mounting procedure followed Noyes (1982) [30]. Specimens were photographed using a Nikon DS-Ri2 camera (Tokyo, Japan) and NIS-Elements Dv4.40 software, attached to a Nikon SMZ18 microscope for overall views and a Nikon Ni microscope for detailed structures of slide-mounted specimens. Body length was measured from specimens before slide-mounting, and all other measurements were taken from slide-mounted specimens. Morphological identification was performed based on photographs of adults and slide-mounted specimens, with reference to the works of Huang and Polaszek [4] and Noyes [2]. All type material and the other specimens examined are deposited in the College of Plant Protection, Fujian Agriculture and Forestry University, Fuzhou, China (FAFU).

### 2.3. DNA Extraction, Library Preparation and Next-Generation Sequencing

Genomic DNA was non-destructively extracted from specimens using the DNeasy Blood and Tissue Kit (QIAGEN GmbH, Hilden, Germany). The wasps remained structurally intact following DNA extraction, and some individuals were used for the preparation of slide-mounted specimens. The extracted DNA was stored at −20 °C, and its concentration was quantified using the Qubit^®^ DNA Assay Kit in a Qubit^®^ 2.0 Fluorometer (Life Technologies, Carlsbad, CA, USA).

A total amount of 1 µg of DNA per sample was used as input to prepare the sequencing libraries. Library construction was performed with the CLEANNGS DNA Kit according to the manufacturer’s protocol, which involved end repairing, A-tailing, and ligation of Illumina adapters. Unique index codes were added to each library for sample multiplexing.

The resulting DNA libraries were sequenced on the Illumina NovaSeq 6000 platform to generate 150 bp paired-end reads.

### 2.4. Mitochondrial Genome Assembly and Annotation

To ensure the reads were reliable and free from artificial bias in downstream analyses, raw data in FASTQ format were processed through a series of quality control (QC) procedures in-house C scripts. QC standards were the following: (1) removing reads with ≥10% unidentified nucleotides (N); (2) removing reads with >50% bases having a Phred quality score < 5; (3) removing reads with >10 bp not aligned to the adapter, allowing ≤10% mismatches; (4) removing putative PCR duplicates generated during the library construction process. After QC, we used MitoZ v2.4 [31], MEGAHIT v1.2.9 [32] and SPAdes v3.10.1 [33] to assemble the clean data and Pilon v1.3.2 [34] to correct the assembled mitochondrial sequence.

The complete mitochondrial genome was annotated using the MITOS Web Server (https://usegalaxy.eu/?tool_id=mitos2, accessed on 20 October 2025) with the invertebrate mitochondrial genetic code [35]. The putative tRNA genes were confirmed by the tRNAscan-SE (http://lowelab.ucsc.edu/tRNAscan-SE/, accessed on 20 October 2025) [36]. For protein-coding genes that were not annotated, the Open Reading Frame (ORF) search function on the NCBI website was used for re-annotation (https://www.ncbi.nlm.nih.gov/, accessed on 20 October 2025). Finally, the full annotation information of the complete mitochondrial genome was obtained.

### 2.5. Mitochondrial Genome Sequence Analysis

The base composition and codon usage of the protein-coding genes were calculated using MEGA12 [37] and Geneious v8.0.4 [38]. The AT-skew and GC-skew were computed according to the formulae: AT-skew = (A% − T%)/(A% + T%) and GC-skew = (G% − C%)/(G% + C%) [39]. We used DnaSP v5 [40] to calculate the ratio of the non-synonymous substitution rate (Ka) to the synonymous substitution rate (Ks) of protein-coding genes and thus analyze the evolutionary rate of *Encarsia* using the mitochondrial genome of *Trichopria drosophilae* (Platygastroidea) as the outgroup. The relative synonymous codon usage (RSCU) of protein-coding genes was calculated by PhyloSuite v1.2.3 [41], and RSCU plots for each species were generated.

### 2.6. Phylogenetic Reconstruction

A total of 13 PCGs from 32 mitochondrial genomes were selected for phylogenetic analysis, including 24 species of other families of Chalcidoidea, 6 *Encarsia* species from Aphelinidae and 2 outgroup species from Cynipoidea: *Trichagalma acutissimae*, and Diapriidae: *Trichopria drosophilae* (Table 1). We extracted protein-coding genes using PhyloSuite v1.2.3 [41]. We aligned the protein-coding genes of these 32 species using the G-INS-I algorithm in MAFFT v7.313 [42], then used Gblocks v0.91b [43] to select conserved sites. Substitution saturation tests were performed on the PCG datasets using PhyloSuite v1.2.3 [41]. ModelFinder v2.2.0 [44] was used to select the optimal evolutionary model. When reconstructing the phylogenetic relationships between Aphelinidae and its allied families, maximum likelihood (ML) analysis was employed. Specifically, we used IQ-TREE v1.6.8 [45] with the GTR+F+I+G4 model and 1000 bootstrap replicates. For Bayesian inference (BI) analysis, we used MrBayes v3.2.6 [46] with the GTR+F+I+G4 model. The analysis was run for 10 million generations, with sampling conducted every 1000 generations, and the burn-in rate of the trees was set at 25%. The phylogenetic tree was edited in iTOL v7.2.2 (https://itol.embl.de, accessed on 25 October 2025).

### 2.7. Terminology, Morphological Measurement and Abbreviations

Terminology and morphological measurement follow Huang (1994) [3] with some modification, and the following abbreviations are used: F1, F2, etc. = funicle antennomeres 1, 2, etc.; C1, C2, etc. = club antennomeres 1, 2, etc.; T1, T2, etc. = gastral tergites 1, 2, etc.

Abbreviations for depositories of specimens are as follows:
FAFUCollege of Plant Protection, Fujian Agriculture and Forestry University, Fuzhou, Fujian, China.IEUNIstituto de Entomologia Agraria, Universita degli Studi di Napoli, Portici, Italy.

## 3. Results

### 3.1. Species Accounts

Huang & Polaszek (1998) [4] provided a detailed species identification key for females of the 72 known species of genus *Encarsia* in China (excluding 4 species known only from males). Based on this key, only the modified key couplets for the 2 newly described species are presented herein for identification and comparison.

#### 3.1.1. Modified Key Couplets to the Species of *Encarsia* (Female) from China by Huang & Polaszek (1998) [4]

Couplet:45Face with one or two brown to dark brown cross bands above the toruli (antennal insertions. Side lobes of mesoscutum each with three setae...........................................................................................46
–
Face uniformly pale. Side lobes of mesoscutum each with two setae................................................50...........................................................................................................................................................................................50Mid-lobe of mesoscutum largely pale.................................................................................................50A
–
Mid-lobe of mesoscutum dark................................................................................................................5150AAntenna yellow except pedicel. Placoid sensilla on scutellum distinctly closely placed, separated by little more than their own maximum diameter................................................................*E. affectata*
–
Antenna with pedicel slightly brown dorsally, F3, F4 pale brown yellow, other segments brown yellow. Placoid sensilla on scutellum distinctly widely placed, separated by clearly more than their own maximum diameter..................................................................................*E. cinnamomi* **sp.n.**

Couplet:68Mid-lobe of mesoscutum with 8 or more setae, and entirely dark. F2 longer than wide and without a longitudinal sensillum......................................................................................................................*E. exserta*
–
Mid-lobe of mesoscutum with 2 or 4 setae. F2 either quadrate or with one longitudinal sensillum...... ......................................................................................................................................................................68A68AMid-lobe of mesoscutum with 4 setae........................................................................................................69
–
Mid-lobe of mesoscutum with 2 setae.........................................................................*E. ophiopogonis* **sp.n.**

#### 3.1.2. *Encarsia cinnamomi* Wang & Huang, **sp.n.** (Figure 1 and Figure 2)

*Diagnosis*. *Encarsia cinnamomi* **sp.n.** resembles *E. affectata* (Silvestri), but can be distinguished from the latter by: (1) antenna with pedicel slightly dark brown dorsally, F3, F4 pale brown yellow, other funiculars brown yellow (*E. affectata*: antenna yellow except pedicel brown); (2) placoid sensilla on scutellum distinctly widely placed, separated by clearly more than their own maximum diameter (*E. affectata*: placoid sensilla on scutellum distinctly closely placed, separated by little more than their own maximum diameter).

**Figure 1:**
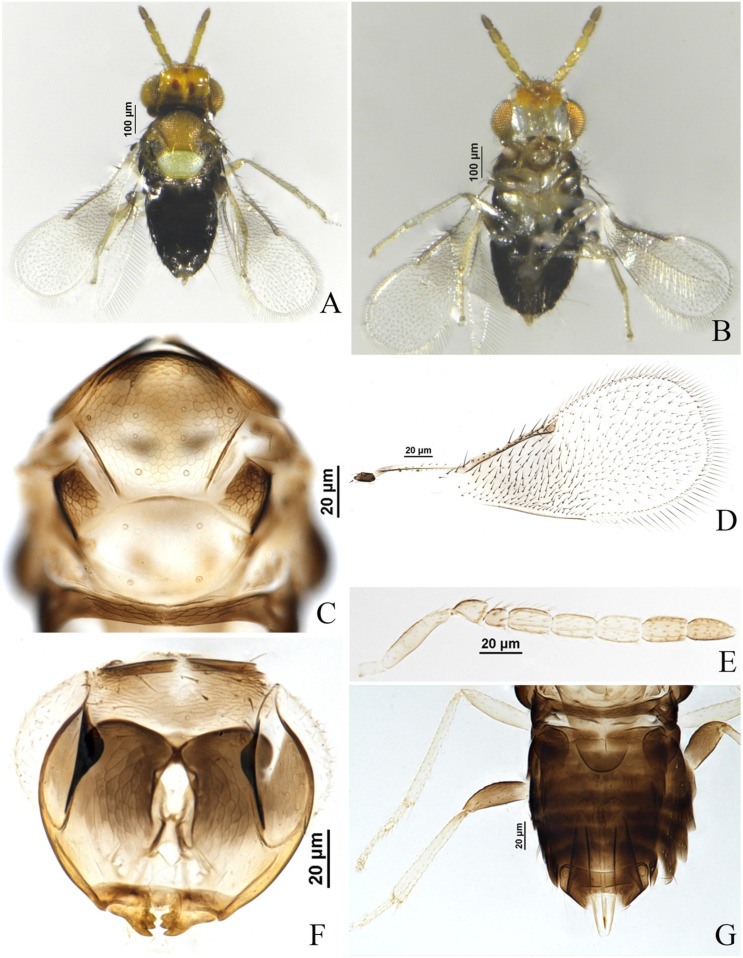
*Encarsia cinnamomi*, **sp.n.** female. (**A**) adult in dorsal view; (**B**) adult in ventral view; (**C**) mesosoma; (**D)** fore wing; (**E**) antennae; (**F**) head; (**G**) metasoma.

**Figure 2:**
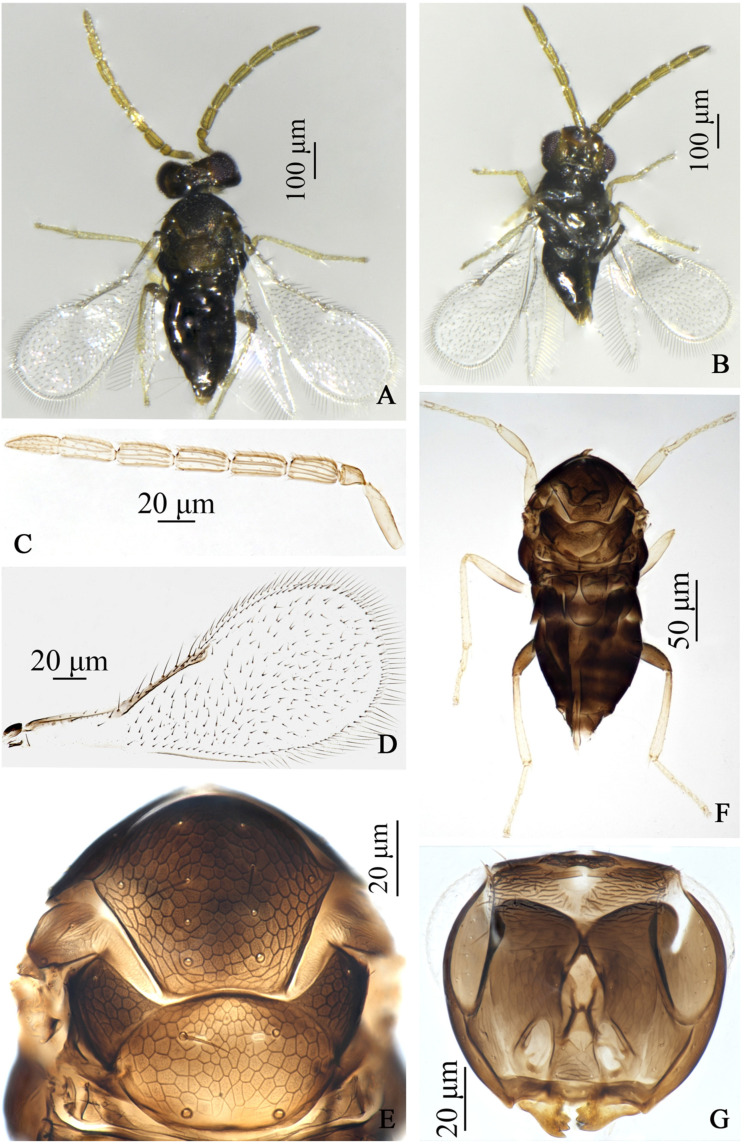
*Encarsia cinnamomi*, **sp.n.** male (FZ). (**A**) adult in dorsal view; (**B**) adult in ventral view; (**C**) antennae; (**D**) forewing; (**E**) mesosoma; (**F**) body; (**G**) head.

*Description*. **Female.** Body length 0.70 mm. **Color.** Head with vertex brown yellow, 2 dark brown spots behind ocellus, face pale, clypeus medially, occiput, mandible apically dark brown; antenna with pedicel slightly dark brown dorsally, F3, F4 pale brown yellow, other funiculars brown yellow; pronotum, axilla mostly, metanotum, propodeum, mesopleuron dark brown, mid-lobe of mesoscutum, parapsides brown yellow and slightly dark anteriorly, scutellum silvery white; abdomen dark brown, T7 pale distally; ovipositor with third valvula dark laterally; fore wing hyaline, area below marginal vein infuscate; legs pale, hind coxae and hind femora dark brown. **Head.** Eyes setose; mandible with 3 acute teeth; antennal formula 1,1,4,2, F1 shortest, F2–F4 approximately equal in length, C2 slightly longer than C1, except F1, other flagellar segments each with 3–4 longitudinal sensilla. **Mesosoma.** Mid-lobe of mesoscutum with 10 setae, each parapside with 2 setae, each axilla with 1 seta; scutellum with 2 pairs of setae, distance between the fore pair of setae approximately equal in length to that between the hind pair of setae, placoid sensilla on scutellum distinctly widely placed, separated by clearly more than their own maximum diameter and close to the fore pair of setae. **Fore wing.** 2.58× as long as broad, marginal fringe 0.26× width of fore wing; submarginal vein with 2 setae, marginal vein with 6–7 setae along anterior margin, basal cell with 2 fine setae. **Leg.** Tarsal formula 5–5–5. **Metasoma**. Ovipositor located basally at T3, 1.12× as long as midtibia, third valvula 0.47× length of second valvifer.

**Male**. Body length 0.68 mm. Similar to female except: body more dark, T7 pale distally; antenna 8-segmented, yellow brown, pedicel shortest, each flagellar segment nearly equal in length, each with 4–5longitudinal sensilla; fore wing hyaline.

*Host*. *Aleurocanthus cinnamomi* Takahashi on *Cinnamomum camphora* Nees ex Wall (Lauraceae).

*Distribution*. China (Fujian).

*Etymology*. The new species name is derived from the name of the host whitefly, *cinnamomi*.

Material. **Holotype** ♀, China: Fujian, Fuzhou, **ex.** *Aleurocanthus cinnamomi* on *Cinnamomum camphora*, 20.v.2022, coll. Zhigang Dong (FAFU); **Paratypes** 1♀1♂, same data as holotype.

#### 3.1.3. *Encarsia ophiopogonis* Wang & Huang, **sp.n.** (Figure 3)

*Diagnosis. Encarsia ophiopogonis* **sp.n.** resembles *E. diaspidicola* (Silvestri), but can be distinguished from the latter by: (1) mid-lobe of mesoscutum with 2 setae (*E. diaspidicola*: with 4 setae); (2) antenna dark yellow brown, except the basal 1/2 of scape pale (*E. diaspidicola*: brown yellow); (3) pedicel 1.20 times as long as F2 (*E. diaspidicola*: pedicel as long as F2); (4) F2 1.25 times as long as F1 (*E. diaspidicola*: F2 1.43 times as long as F1).

**Figure 3:**
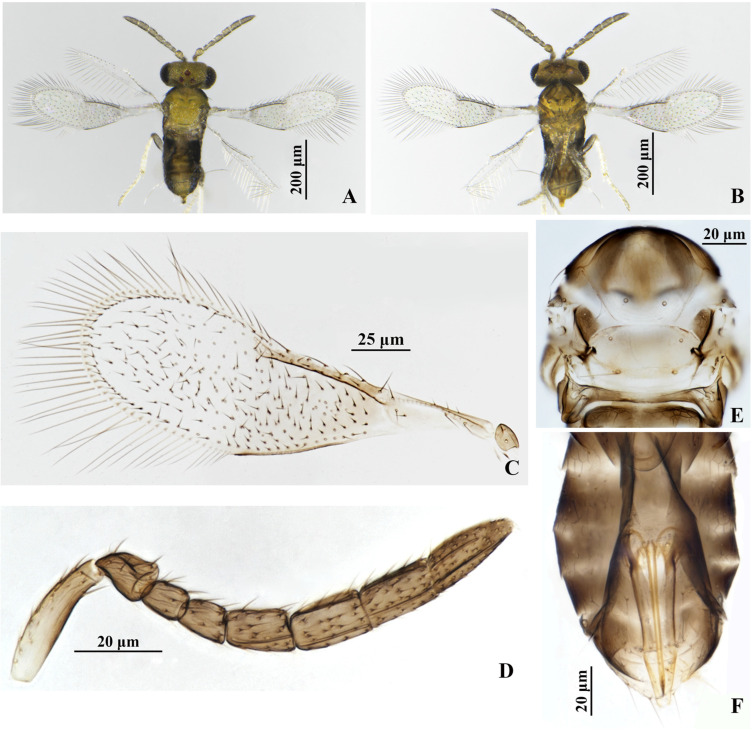
*Encarsia ophiopogonis*, **sp.n.** female. (**A**) adult in dorsal view; (**B**) adult in ventral view; (**C**) fore wing; (**D**) antenna; (**E**) mesosoma; (**F**) metasoma.

*Description*. **Female**. Body length 0.50 mm. **Color**. Head yellow, except clypeus, hind head brown to dark brown; antenna dark yellow brown, except the basal 1/2 of scape pale; mesosomal terga yellow, except pronotum, mid-lobe of mesoscutum anteriorly, axilla, mesopleuron, propodeum brown; petiole, metasomal tergites brown, T7 yellow distally; ovipositor with third valvula pale; fore wing hyaline, area below marginal vein faintly infuscate; legs pale, hind coxae and hind femora dark brown. **Head.** Eyes setose; mandible with 3 acute teeth; antennal formula 1,1,3,3, F1 shortest, F2 slightly shorter than F3, C1 nearly equal to C2 in length, shorter than C3; except F1, other flagellar segments each with 1–2 longitudinal sensilla. **Mesosoma**. Mid-lobe of mesoscutum with 2 setae, each parapside with 1 seta, each axilla with 1 seta; scutellum with 2 pairs of setae, distance between the fore pair of setae longer than that between the hind pair of setae, placoid sensilla on scutellum distinctly widely placed and close to the fore pair of setae. **Fore wing**. 3.38× as long as broad, marginal fringe 0.71× width of fore wing; submarginal vein with 2 setae, marginal vein with 5 setae along anterior margin. **Leg.** tarsal formula 5–5–5. **Metasoma**. Ovipositor located basally at T4, 0.84× as long as midtibia, third valvula 0.50× length of second valvifer.

**Male**. Unknown.

*Host*. *Pinnaspis aspidistrae* (Signoret) on *Ophiopogon japonicus* (L. f.) Ker Gawl.

*Distribution*. China (Fujian).

*Etymology*. The new species name is named after the genus of host plant, *Ophiopogon*.

Material. **Holotype** ♀, China: Fujian, Fuzhou, Fujian Agriculture and Forestry University, **ex.** *Pinnaspis aspidistrae (Signoret) on Ophiopogon japonicus* (L. f.) Ker Gawl., 30.v.2024, coll. Ye Luo and Zhigang Dong (FAFU). **Paratypes** 2♀, same data as holotype (FAFU).

Remarks. The new species resembles *Encarsia diaspidicola* (Silvestri) but distinguished from the latter mainly by setation on the mid-lobe of mesoscutum, as mentioned above. However, it now appears that some errors exist in the description of *E. diaspidicola* by Huang & Polaszek (1998) [4]. At that time, Huang & Polaszek (1998) [4] examined the types of *E. diaspidicola* in the IEUN, described the mid-lobe of mesoscutum with 1+1 or 2+2 setae, and pointed out that the setation on the mid-lobe of mesoscutum varies considerably, from 2 setae (1♀), 1+2 setae (5♀), 2+2 setae (5♀) to 1+2+2 setae. Obviously, most of the types are with 4 setae and variation of 4 setae on the mid-lobe of mesoscutum. Moreover, the 4 setae on the mid-lobe of mesoscutum was originally described in *E. diaspidicola* sp.n. by Silvestri (1909) [48]. So *E. diaspidicola* should have 4 setae on the mid-lobe of mesoscutum, and the Figure 76 in Huang & Polaszek (1998) [4] should be to illustrate the feature of 4 setae instead of 2 setae on the mid-lobe of mesoscutum.

### 3.2. Three Mitochondrial Genomes of Encarsia

#### 3.2.1. Mitochondrial Genome Base Composition and Sequence Signature

**Figure 4:**
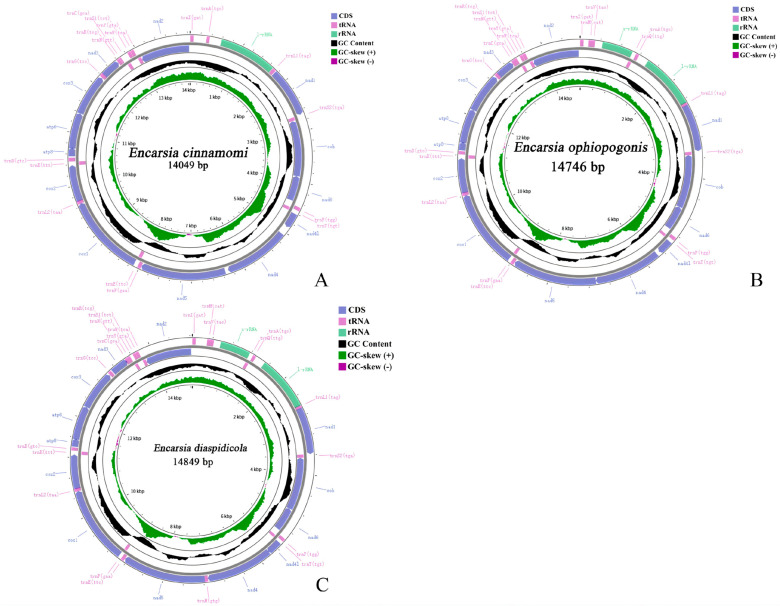
Maps of the sequenced mitogenome. (**A**): *Encarsia cinnamomi*, **sp.n.**; (**B**): *E. ophiopogonis*, **sp.n.**; (**C**): *E. diaspidicola*.

The complete mitochondrial genomes of *Encarsia cinnamomi*, **sp.n.**, *E. ophiopogonis*, **sp.n.** and *E. diaspidicola* are 14,049 bp, 14,746 bp and 14,849 bp in length, respectively (Table A1, Table A2 and Table A3). The mitogenome of *E. cinnamomi*, **sp.n.** comprises 32 genes (13 PCGs, 18 tRNAs, and 1 rRNAs), with 23 genes encoded on the heavy strand (J-strand) and 9 on the light strand (N-strand). The mitogenome of *E. ophiopogonis*, **sp.n.** and *E. diaspidicola* contains the full complement of 37 genes (13 PCGs, 22 tRNAs, and 2 rRNAs), with 27 genes on the J-strand and 10 on the N-strand. Circular maps illustrating the gene arrangements of both mitogenomes are presented in Figure 4A–C. Both genomes exhibit a strong A + T bias, with overall A + T contents of 84.1%, 84.7% and 84.8%, respectively (Table 2). Analysis of the protein-coding genes (PCGs) reveals that the third codon position has the highest A + T content (94.2%, 95.4%, 95.3%), while the second codon position has the lowest (75.2%, 75.4%, 75.6%) (Table 2).

#### 3.2.2. Protein-Coding Gene

**Figure 5:**
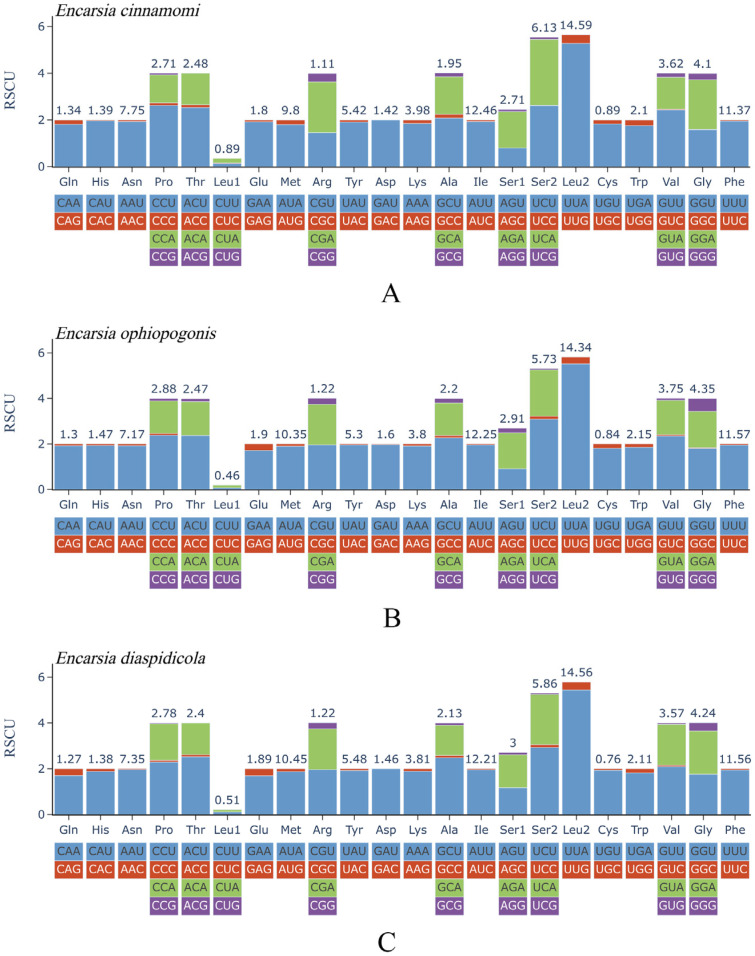
Relative synonymous codon usage of the protein-coding genes. (**A**): *Encarsia cinnamomi*, **sp.n.**; (**B**): *E. ophiopogonis*, **sp.n.**; (**C**): *E. diaspidicola*.

The combined lengths of the 13 protein-coding genes (PCGs) are 10,929 bp in *E. cinnamomi*, 11,136 bp in *E. ophiopogonis* and 11,137 bp in *E. diaspidicola*, with A + T contents of 82.7%, 83.3% and 83.5%, respectively (Table 2). All PCGs in both species use ATN as the start codon. In *E. cinnamomi* and *E. ophiopogonis*, all 13 PCGs are terminated with a TAA stop codon. Most PCGs in *E. diaspidicola* also use TAA, except for *nad3* and *nad4*, which ended with an incomplete termination codon TA and T respectively (Table A1, Table A2 and Table A3). Analysis of relative synonymous codon usage (RSCU) reveals that UUA (Leu2) is the most frequent codon in both species, with RSCU values of 5.28, 5.52 and 5.44, respectively (Figure 5A–C).

The evolutionary rates of the 13 protein-coding genes (PCGs) in three *Encarsia* species were assessed by calculating the non-synonymous to synonymous substitution rate ratio(Ka/Ks), using *Trichopria drosophilae* as a reference. In *E. cinnamomi* **sp.n.**, the Ka/Ks ratios of seven genes were greater than 1; in *E. ophiopogonis* **sp.n.**, those of nine genes exceeded 1; and in *E. diaspidicola*, eight genes had Ka/Ks ratios above 1, indicating that these genes were subject to positive selection. Among them, *nad2* exhibited the highest evolutionary rate in *E. cinnamomi* **sp.n.** and *E. diaspidicola* (Ka/Ks = 2.1642 and 2.8173, respectively), while *atp8* showed the highest evolutionary rate in *E. ophiopogonis* **sp.n.** (Ka/Ks = 3.1021). Notably, *cox1* exhibited the lowest Ka/Ks values in the three species (0.5146, 0.4185 and 0.4070, respectively), indicating it is under strong purifying selection (Table 3).

#### 3.2.3. Gene Characteristics of tRNAs and rRNAs

**Figure 6:**
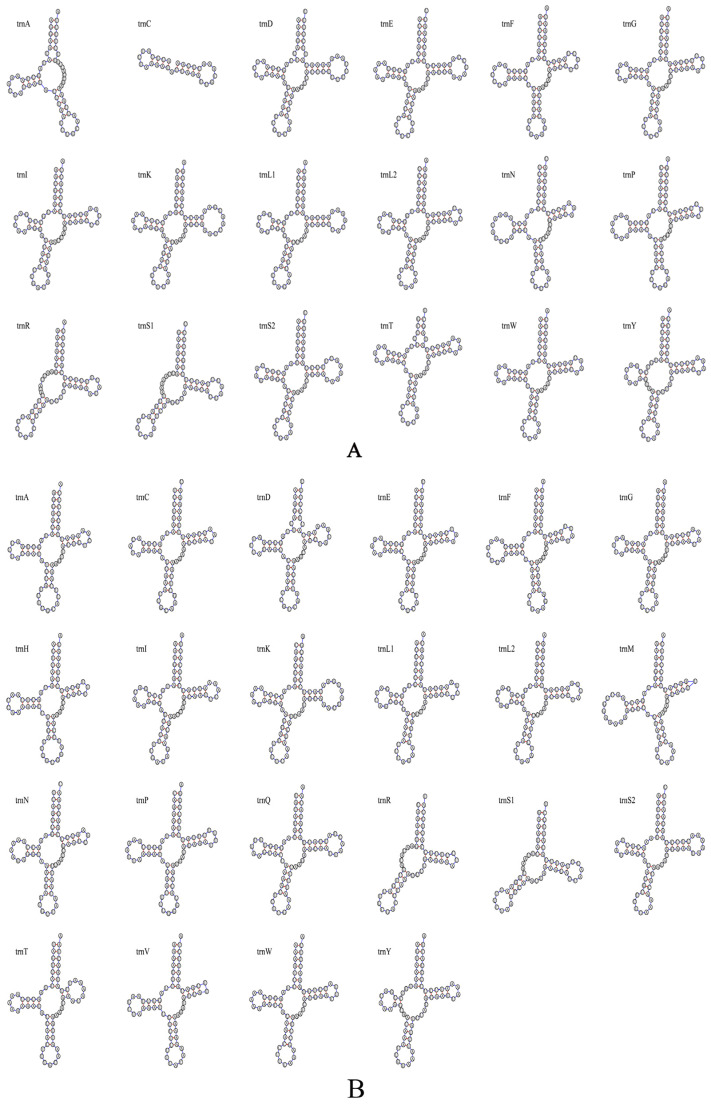

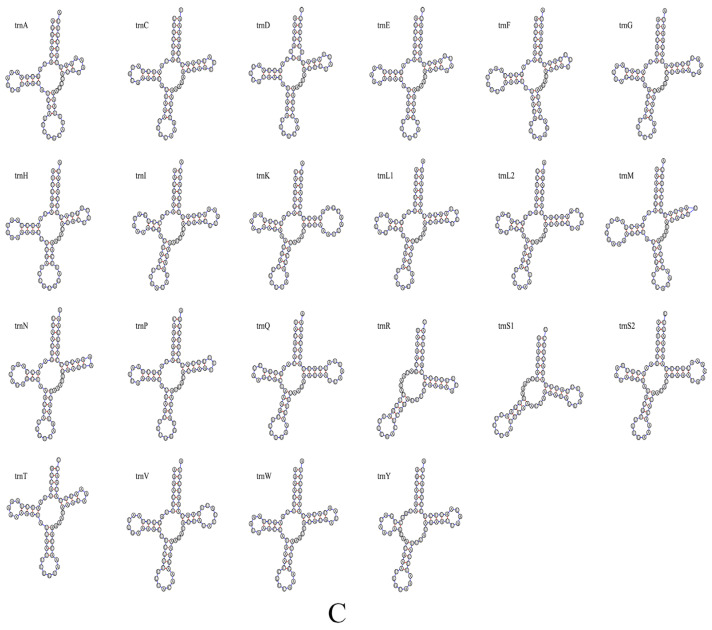
Secondary structure of tRNA gene. (**A**): *Encarsia cinnamomi*, **sp.n.**; (**B**): *E. ophiopogonis*, **sp.n.**; (**C**): *E. diaspidicola*.

The mitochondrial genome of *Encarsia cinnamomi*, **sp.n.** contains 18 tRNA genes, ranging from 28 to 71 bp in length. Most tRNAs exhibit the typical cloverleaf structure except for *trnA*, *trnR* and *trnS1*, which lack the DHU arm, and *trnC*, which is severely truncated, retaining only the anticodon loop and DHU arm. A total of 9 G-U mismatches are identified in its tRNA structures (Figure 6A). In comparison, the mitogenomes of *E. ophiopogonis*, **sp.n.** and *E. diaspidicola* contains 22 tRNA genes, ranging from 55 to 70 bp in length. All tRNAs form the typical cloverleaf structure, with the exception of *trnA* and *trnS1*, both of which lack the DHU arm. Fourteen and ten G-U mismatches are identified in *E. ophiopogonis*, **sp.n.** and *E. diaspidicola* respectively (Figure 6B,C).

Regarding rRNA genes, *E. cinnamomi*, **sp.n.** possesses a single rRNA gene with a length of 1086 bp, representing 7.7% of the complete mitochondrial genome and an A + T content of 86.2% (Table 2). *E. ophiopogonis*, sp.n. and *E. diaspidicola* contain two rRNA genes totaling 1888 bp and 2029 bp, which account for 12.8% and 13.7% of the mitogenome respectively. The small ribosomal RNA gene (*rrnS)* is located between *trnV* and *trnQ*, measuring 606 bp and 750 bp with A + T contents of 87.3% and 88.5%, while the large ribosomal RNA gene (*rrnL)* is situated between *trnA* and *trnL1*, spanning 1282 bp and 1279 bp with A + T contents of 88.2% and 87.9% respectively (Table 2).

#### 3.2.4. Phylogenetic Analysis

**Figure 7:**
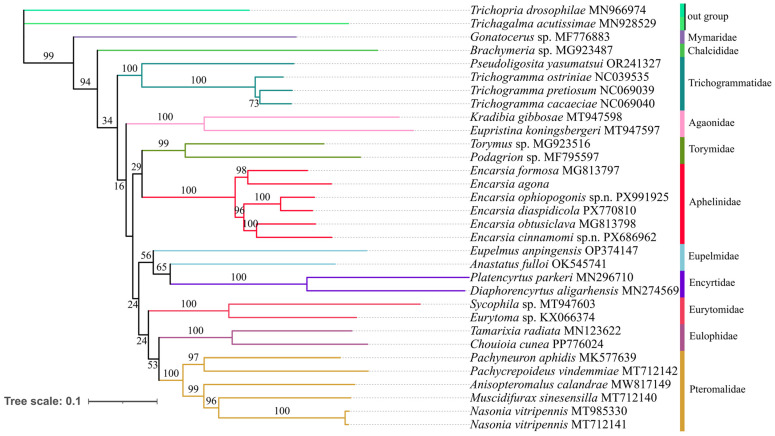
Phylogenetic tree of Aphelinidae and other families of Chalcidoidea inferred by ML analysis based on 13 PCGs from 30 species. Numbers at the nodes are bootstrap values.

**Figure 8:**
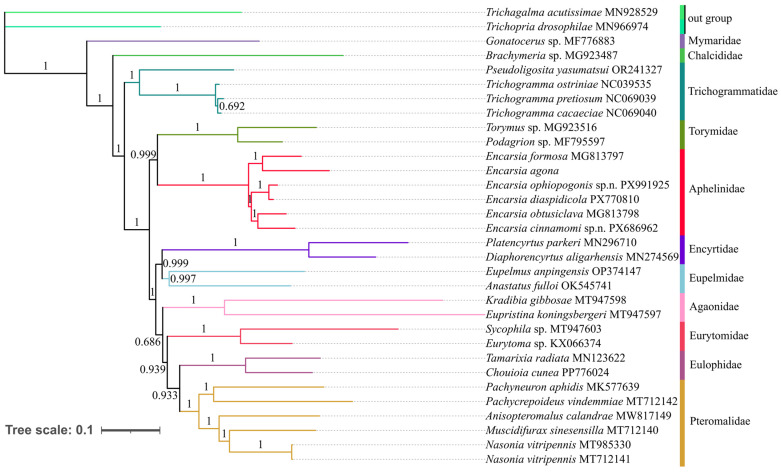
Phylogenetic tree of Aphelinidae and other families of Chalcidoidea inferred by BI analysis based on 13 PCGs from 30 species. Numbers at the nodes are posterior probabilities.

The phylogenetic relationships within Aphelinidae and other families of Chalcidoidea were reconstructed based on three newly sequenced mitochondrial genomes of *Encarsia*, along with 27 previously recorded mitogenomes in GenBank. Phylogenetic analyses were conducted using both concatenated PCG123 datasets under Bayesian inference (BI) and maximum likelihood (ML) frameworks (Figure 7 and Figure 8). In both ML and BI phylogenetic analyses, *Encarsia* was supported as a monophyletic group, in which *E. ophiopogonis*, **sp.n.** and *E. diaspidicola* clustered in the same clade with a bootstrap value of 100%. This indicates a close phylogenetic affinity between the two species, which are also extremely similar in morphology. The new species *E. cinnamomi* **sp.n.** and *E. obtusiclava* formed a sister relationship. Meanwhile, the monophyly of each family within Chalcidoidea was strongly supported.

However, our results regarding the backbone relationships among Chalcidoidea families require cautious interpretation. The ML and BI analyses yielded incongruent topologies for interfamilial relationships (Figure 7 and Figure 8), and crucially, these deeper nodes received consistently low statistical support (e.g., <95% bootstrap) in the ML analysis. This lack of robust resolution, combined with conflicts with more comprehensive phylogenomic studies of Chalcidoidea, precludes drawing definitive conclusions about interfamilial relationships from the current dataset. Despite these limitations, our findings demonstrate the potential of mitogenomic data for resolving relationships within Chalcidoidea. They also underscore that substantially increased taxon sampling, particularly at the family level, is a critical prerequisite for confidently resolving the superfamily’s backbone phylogeny.

## 4. Discussion

*Encarsia ophiopogonis* Wang & Huang, **sp.n.**, resembles *E. diaspidicola* (Silvestri) but is distinguished from the latter mainly by the setation on the mid-lobe of mesoscutum and antennal characteristics, as well as distinct interspecific genetic distances in the mitochondrial genome. The sequence identity of the mitochondrial genomes between these two species is 92.20%, and the genetic distance reaches 7.62%, which has reached the interspecific genetic level. It is generally considered that an insect genetic distance greater than 3% reaches the interspecific level [49]. *E. diaspidicola* was originally described by Silvestri (1909) [48] with 4 setae on the mid-lobe of mesoscutum. However, it now appears that some errors exist in the description of *E. diaspidicola* by Huang & Polaszek (1998) [4], in which Figure 76 should illustrate an individual with 4 setae instead of 2 setae on the mid-lobe of mesoscutum, although there is variation of the setae number on the mid-lobe of mesoscutum.

The mitochondrial genome sequence serves as a pivotal molecular marker that is frequently employed in phylogenetic studies to clarify the evolutionary relationships among closely related insect taxa [50,51]. The mitochondrial genomes of the three *Encarsia* species characterized in this study range from 14,049 to 14,849 bp in length. This length range is comparable to that of the mitochondrial genomes previously reported for *Encarsia* [27,47], Eupelmidae [52], and Pteromalidae [53], but is consistently shorter than the previously recorded lengths. This discrepancy might be attributable to unsequenced regions in the three mitochondrial genomes analyzed herein. For instance, only 32 genes were identified in *E*. *cinnamomi*, **sp.n.**, with 4 tRNA genes and 1 rRNA gene undetected; additionally, the secondary structures of trnC and trnA were found to be incomplete. This may be caused by incomplete sequencing. For *E. ophiopogonis*, sp.n. and *E. diaspidicola*, although they contain all 37 genes of the mitochondrial genome, their relatively short length may be attributed to incomplete sequencing of the non-coding regions, whereas *E. formosa* [27] possesses a considerably long non-coding region. Usually, such species have a high AT content, which makes sequencing more difficult. In Chalcidoidea (Hymenoptera), the AT content typically falls within the range of 75% to 90% [54]. The AT content of these three species described in this study was determined to be 84.1% to 84.8%, which conforms to this typical feature. The mitochondrial genome of *E*. *cinnamomi*, **sp.n.** harbors 23 genes on the J-strand and 9 genes on the N-strand, while those of *E. ophiopogonis*, **sp.n.** and *E. diaspidicola* contain 27 genes on the J-strand and 10 genes on the N-strand. This gene distribution pattern aligns with the general observation across numerous species, in which the majority of coding genes are situated on the J-strand, with only a small proportion on the N-strand [20]. These three species reported in this study all exhibit negative AT-skew and positive GC-skew. This is opposite to the typical pattern of nucleotide composition bias in insect mitochondrial genomes, which is positive AT-skew and negative GC-skew [55], but is consistent with the patterns observed in some previous reports [52,56]. Typically, *trnS1* in animals lacks the DHU arm and thus does not possess the canonical cloverleaf structure [20]. The secondary structures of most tRNAs in the three new species are canonical cloverleaf structures, but some gene structural deletions were also observed. In addition to the absence of the DHU arm in *trnS1* across all three species, the DHU arm is also missing in *trnR*; this is consistent with the tRNA secondary structure of the recently reported mitochondrial genome of *Encarsia agona* [47]; furthermore, the *trnC* gene of *E*. *cinnamomi*, **sp.n.** has a severe structural deletion.

In the phylogenetic analysis, *E. cinnamomi*, **sp.n.,** *E. ophiopogonis*, **sp.n.** and *E. diaspidicola* are well separated from the other three known species. Meanwhile, in another study, the single gene *cox1* could not separate the species well or reflect the phylogenetic relationships within Aphelinidae [57], which indicates that the mitochondrial genome has great application potential in species identification of Aphelinidae. In both ML and BI phylogenetic analysis, Aphelinidae forms a sister group with Torymidae, which is different from the findings of He et al. (2025) [58], Xing et al. (2021) [59] and Zhao et al. (2021) [60] where it forms a sister group to Trichogrammatidae, and also differs from the findings of Zhu et al. (2018) [27], where it forms a sister group to (Trichogrammatidae + Agaonidae). Morphologically, Aphelinidae is generally close to Encyrtidae and Eulophidae. However, the phylogenetic analysis indicates that it is more closely related to Torymidae. In a recent study, the authors investigated the phylogenetic relationships of Chalcidoidea based on the exons (AHE414) and UCE datasets, and their results recovered Aphelinidae as the sister group to (Encyrtidae + Eunotidae) [61]. Currently, only six mitochondrial genomes of *Encarsia* are available worldwide (including the three species presented in this study), and mitochondrial genomic data for other genera of Aphelinidae remain entirely absent. To further clarify the phylogenetic relationships within Aphelinidae and among all families of Chalcidoidea, future studies should prioritize expanding taxon sampling for mitochondrial genomic sequencing across these groups.

## Figures and Tables

**Table 1:** Mitogenomes of Aphelinidae, other families within the Chalcidoidea and outgroups used in this study.

Family		Species	Accession Number	Source
Aphelinidae		*Encarsia formosa*	MG813797	GenBank
		*Encarsia obtusiclava*	MG813798	GenBank
		*Encarsia agona*	-	[47]
		*Encarsia cinnamomi* **sp.n.**	PX686962	This manuscript
		*Encarsia ophiopogonis* **sp.n.**	PX991925	This manuscript
		*Encarsia diaspidicola*	PX770810	This manuscript
Mymaridae		*Gonatocerus* sp.	MF776883	GenBank
Chalcididae		*Brachymeria* sp.	MG923487	GenBank
Trichogrammatidae		*Trichogramma ostriniae*	NC039535	GenBank
		*Trichogramma pretiosum*	NC069039	GenBank
		*Trichogramma cacaeciae*	NC069040	GenBank
		*Pseudoligosita yasumatsui*	OR241327	GenBank
Agaonidae		*Kradibia gibbosae*	MT947598	GenBank
		*Eupristina koningsbergeri*	MT947597	GenBank
Torymidae		*Torymus* sp.	MG923516	GenBank
		*Podagrion* sp.	MF795597	GenBank
Eupelmidae		*Eupelmus anpingensis*	OP374147	GenBank
		*Anastatus fulloi*	OK545741	GenBank
Eulophidae		*Chouioia cumea*	PP776024	GenBank
		*Tamarixia radiata*	MN123622	GenBank
Pteromalidae		*Pachyneuron aphidis*	MK577639	GenBank
		*Pachycrepoideus vindemmiae*	MT712142	GenBank
		*Anisopteromalus calandrae*	MW817149	GenBank
		*Muscidifurax sinesensilla*	MT712140	GenBank
		*Nasonia vitripennis*	MT985330	GenBank
		*Nasonia vitripennis*	MT712141	GenBank
Encyrtidae		*Platencyrtus parkeri*	MN296710	GenBank
		*Diaphorencyrtus aligarhensis*	MN274569	GenBank
Eurytomidae		*Sycophila* sp.	MT947603	GenBank
		*Eurytoma* sp.	KX037466	GenBank
outgroop	Cynipidae	*Trichagalma acutissimae*	MN928529	GenBank
	Diapriidae	*Trichopria drosophilae*	MN966974	GenBank

**Table 2:** Nucleotide composition of the whole mitogenomes of *Encarsia cinnamomi*, **sp.n.**, *E. ophiopogonis*, **sp.n.** and *E. diaspidicola*.

Species	Feature	Length bp	A %	T (U) %	C %	G %	AT %	AT-Skew	GC-Skew
*E. cinnamomi*, **sp.n.**	Whole genome	14,049	36.5	47.5	5.9	9.9	84.1	−0.131	0.253
	Protein-coding genes	10,929	35.3	47.3	6.6	10.6	82.7	−0.145	0.233
	1st codon site	3643	38.0	40.5	7.6	13.9	78.5	−0.033	0.293
	2nd codon site	3643	22.9	52.3	13.4	11.4	75.2	−0.391	−0.077
	3rd codon site	3643	42.1	52.1	1.6	3.9	94.2	−0.107	0.417
	tRNA genes	1142	41.6	47.0	4.3	7.1	88.6	−0.061	0.246
	rRNA genes	1086	39.5	46.5	4.8	9.0	86.2	−0.081	0.304
*E. ophiopogonis*, **sp.n.**	Whole genome	14,746	36.9	47.8	6.0	9.3	84.7	−0.129	0.216
	Protein-coding genes	11,136	35.8	47.5	6.6	10.1	83.3	−0.140	0.209
	1st codon site	3712	39.1	40.0	7.2	13.7	79.1	−0.011	0.308
	2nd codon site	3712	22.6	52.8	13.2	11.4	75.4	−0.400	−0.074
	3rd codon site	3712	42.9	52.5	1.5	3.2	95.4	−0.100	0.376
	tRNA genes	1426	42.8	46.1	4.4	6.7	88.9	−0.037	0.207
	rRNA genes	1888	38.5	49.5	4.2	7.8	87.9	−0.125	0.298
*E. diaspidicola*	Whole genome	14,849	36.7	48.0	6.0	9.3	84.8	−0.133	0.216
	Protein-coding genes	11,137	35.8	47.7	6.6	9.9	83.5	−0.143	0.200
	1st codon site	3713	39.1	40.5	7.1	13.3	79.6	−0.018	0.304
	2nd codon site	3713	22.9	52.7	13.1	11.3	75.6	−0.394	−0.074
	3rd codon site	3713	42.8	52.5	1.5	3.2	95.3	−0.102	−0.362
	tRNA genes	1449	42.0	47.1	4.3	6.6	89.1	−0.057	0.211
	rRNA genes	2029	38.2	49.9	4.1	7.8	88.1	−0.133	0.311

**Table 3:** Ka/Ks values of the mitochondrial genome protein-coding genes of *Encarsia cinnamomi*, **sp.n.**, *E. ophiopogonis*, **sp.n.** and *E. diaspidicola*.

	Ka			Ks			Ka/Ks		
Species	E. C.	E. O.	E. D.	E. C.	E. O.	E. D.	E. C.	E. O.	E. D.
*nad1*	0.3689	0.3838	0.3800	0.3702	0.3207	0.2927	0.9965	1.1968	1.2983
*cob*	0.2417	0.2226	0.2176	0.4383	0.4088	0.3873	0.5514	0.5445	0.5168
*nad6*	0.5721	0.5085	0.4745	0.3403	0.2408	0.4232	1.6812	2.1117	1.1212
*nad4L*	0.6574	0.5828	0.5456	0.3245	0.2136	0.2860	2.0259	2.7285	1.9077
*nad4*	0.3796	0.3886	0.3967	0.3290	0.2818	0.2914	1.1538	1.3790	1.3614
*nad5*	0.3705	0.3936	0.3931	0.2845	0.2886	0.3110	1.3023	1.3638	1.2640
*cox1*	0.1657	0.1590	0.1540	0.3220	0.3799	0.3784	0.5146	0.4185	0.4070
*cox2*	0.3210	0.3202	0.3141	0.3858	0.2995	0.3385	0.8320	1.0691	0.9279
*atp8*	0.4914	0.4374	0.4299	0.2965	0.1410	0.1674	1.6573	3.1021	2.5681
*atp6*	0.3179	0.3090	0.3221	0.3913	0.4293	0.3975	0.8124	0.7198	0.8103
*cox3*	0.3840	0.3868	0.3866	0.4099	0.4095	0.3875	0.9368	0.9446	0.9977
*nad3*	0.4193	0.3891	0.3877	0.2609	0.2799	0.2863	1.6071	1.3901	1.3542
*nad2*	0.5363	0.5607	0.5429	0.2478	0.2026	0.1927	2.1642	2.7675	2.8173

Notes: E. C.: *Encarsia cinnamomi*, **sp.n.**; E. O.: *E. ophiopogonis*, **sp.n.**; E. D.: *E. diaspidicola*.

## Data Availability

Data are contained within the article.

## Appendix A

**Table A1:** Characteristics of the mitochondrial genome of *Encarsia cinnamomi*, **sp.n.**

Gene	Strand	Position (bp)	Length (bp)	Start Codons	Stop Codons	Intergenic Sequence (bp)
*trnI*	forward	1–67	67			242
*trnA*	forward	310–366	57			225
*rrnL*	forward	592–1677	1086			5
*trnL1*	forward	1683–1750	68			12
*nad1*	forward	1763–2689	927	ATG	TAA	−1
*trnS2*	reverse	2689–2755	67			0
*cob*	reverse	2756–3898	1143	ATG	TAA	−1
*nad6*	reverse	3898–4404	507	ATT	TAA	66
*trnP*	forward	4471–4538	68			4
*trnT*	reverse	4543–4604	62			10
*nad4L*	forward	4615–4902	288	ATT	TAA	158
*nad4*	forward	5061–6296	1236	ATG	TAA	57
*nad5*	forward	6354–7970	1617	ATT	TAA	−1
*trnF*	forward	7970–8036	67			0
*trnE*	reverse	8037–8105	69			8
*cox1*	forward	8114–9652	1539	ATG	TAA	−5
*trnL2*	forward	9648–9712	65			−12
*cox2*	forward	9701–10,387	687	ATA	TAA	2
*trnK*	reverse	10,390–10,459	70			3
*trnD*	forward	10,463–10,532	70			25
*atp8*	forward	10,558–10,719	162	ATT	TAA	−13
*atp6*	forward	10,707–11,384	678	ATG	TAA	−1
*cox3*	forward	11,384–12,169	786	ATG	TAA	6
*trnG*	forward	12,176–12,241	66			−3
*nad3*	forward	12,239–12,592	354	ATA	TAA	3
*trnR*	forward	12,596–12,653	58			3
*trnC*	forward	12,657–12,687	31			32
*trnN*	reverse	12,720–12,786	67			46
*trnS1*	forward	12,833–12,891	59			4
*trnY*	forward	12,896–12,960	65			−2
*trnW*	reverse	12,959–13,024	66			−2
*nad2*	reverse	13,023–14,027	1005	ATT	TAA	22

Notes: *trnM*, *trnV*, *trnQ*, *trnH* and *rrnS* not annotated.

**Table A2:** Characteristics of the mitochondrial genome of *Encarsia ophiopogonis*, **sp.n.**

**Gene**	**Strand**	**Position (bp)**	**Length (bp)**	**Start Codons**	**Stop Codons**	**Intergenic** **Sequence (bp)**
*trnI*	forward	1–67	67			96
*trnM*	forward	164–232	69			−1
*trnV*	forward	232–294	63			146
*rrnS*	forward	441–1046	606			2
*trnQ*	reverse	1049–1117	69			6
*trnA*	forward	1124–1187	64			204
*rrnL*	forward	1392–2486	1095			−17
*trnL1*	forward	2470–2534	65			−6
*nad1*	forward	2529–3464	936	ATA	TAA	−2
*trnS2*	reverse	3463–3529	67			−2
*cob*	reverse	3528–4670	1143	ATG	TAA	−1
*nad6*	reverse	4670–5176	507	ATT	TAA	70
*trnP*	forward	5247–5314	68			2
*trnT*	reverse	5317–5375	59			6
*nad4L*	forward	5382–5669	288	ATT	TAA	−7
*nad4*	forward	5663–7000	1332	ATG	TAA	−2
*trnH*	forward	6999–7063	65			0
*nad5*	forward	7064–8734	1671	ATT	TAA	5
*trnF*	forward	8740–8806	67			−1
*trnE*	reverse	8806–8870	65			0
*cox1*	forward	8871–10,409	1539	ATA	TAA	−5
*trnL2*	forward	10,405–10,470	66			−12
*cox2*	forward	10,459–11,142	684	ATA	TAA	0
*trnK*	reverse	11,143–11,212	70			5
*trnD*	forward	11,218–11,281	64			17
*atp8*	forward	11,299–11,460	162	ATT	TAA	−13
*atp6*	forward	11,448–12,125	678	ATG	TAA	−1
*cox3*	forward	12,125–12,910	786	ATG	TAA	9
*trnG*	forward	12,920–12,985	66			−3
*nad3*	forward	12,983–13,336	354	ATA	TAA	0
*trnR*	forward	13,337–13,391	55			−1
*trnC*	forward	13,391–13,455	65			1
*trnN*	reverse	13,457–13,520	64			1
*trnS1*	forward	13,522–13,578	57			4
*trnY*	forward	13,583–13,647	65			−2
*trnW*	reverse	13,646–13,711	66			−2
*nad2*	reverse	13,710–14,714	1005	ATT	TAA	32

**Table A3:** Characteristics of the mitochondrial genome of *Encarsia diaspidicola*.

Gene	Strand	Position (bp)	Length (bp)	Start Codons	Stop Codons	Intergenic Sequence (bp)
*trnI*	forward	1–67	67			226
*trnM*	forward	294–360	67			−1
*trnV*	forward	360–427	68			138
*rrnS*	forward	428–1177	750			7
*trnQ*	reverse	1179–1246	68			−1
*trnA*	forward	1246–1308	63			203
*rrnL*	forward	1309–2587	1279			−17
*trnL1*	forward	2589–2653	65			−6
*nad1*	forward	2648–3586	939	ATA	TAA	−2
*trnS2*	reverse	3585–3652	68			−2
*cob*	reverse	3651–4793	1143	ATG	TAA	−1
*nad6*	reverse	4793–5365	573	ATG	TAA	70
*trnP*	forward	5370–5434	65			2
*trnT*	reverse	5437–5495	59			1
*nad4L*	forward	5497–5784	288	ATT	TAA	−7
*nad4*	forward	5778–7116	1339	ATG	T	−3
*trnH*	forward	7114–7178	65			0
*nad5*	forward	7179–8849	1671	ATT	TAA	3
*trnF*	forward	8853–8918	66			0
*trnE*	reverse	8919–8982	64			3
*cox1*	forward	8986–10,524	1539	ATG	TAA	−5
*trnL2*	forward	10,520–10,586	67			−12
*cox2*	forward	10,587–11,258	672	ATT	TAA	0
*trnK*	reverse	11,259–11,328	70			4
*trnD*	forward	11,333–11,396	64			15
*atp8*	forward	11,412–11,573	162	ATT	TAA	−13
*atp6*	forward	11,570–12,238	669	ATA	TAA	−1
*cox3*	forward	12,238–13,023	786	ATG	TAA	9
*trnG*	forward	13,033–13,099	67			18
*nad3*	forward	13,100–13,449	353	ATT	TA	−20
*trnR*	forward	13,450–13,504	55			−1
*trnC*	forward	13,504–13,566	63			0
*trnN*	reverse	13,567–13,631	65			0
*trnS1*	forward	13,632–13,688	57			1
*trnY*	forward	13,690–13,755	66			−2
*trnW*	reverse	13,754–13,819	66			−2
*nad2*	reverse	13,818–14,825	1008	ATA	TAA	24
